# Biosynthesis of the Novel Endogenous 15-Lipoxygenase Metabolites *N*-13-Hydroxy-octodecadienoyl-ethanolamine and 13-Hydroxy-octodecadienoyl-glycerol by Human Neutrophils and Eosinophils

**DOI:** 10.3390/cells10092322

**Published:** 2021-09-05

**Authors:** Anne-Sophie Archambault, Francesco Tinto, Élizabeth Dumais, Volatiana Rakotoarivelo, Magdalena Kostrzewa, Pier-Luc Plante, Cyril Martin, Mélissa Simard, Cristoforo Silvestri, Roxane Pouliot, Michel Laviolette, Louis-Philippe Boulet, Rosa Maria Vitale, Alessia Ligresti, Vincenzo Di Marzo, Nicolas Flamand

**Affiliations:** 1 Centre de Recherche de l’Institut Universitaire de Cardiologie et de Pneumologie de Québec, Faculté de Médecine, Université Laval, Québec City, QC G1V 4G5, Canada; Anne-Sophie.archambault@criucpq.ulaval.ca (A.-S.A.); FrancescoTinto88@gmail.com (F.T.); Elizabeth.Dumais@criucpq.ulaval.ca (É.D.); Volatiana.Rakotoarivelo@criucpq.ulaval.ca (V.R.); cyril.martin@criucpq.ulaval.ca (C.M.); Melissa.Simard@criucpq.ulaval.ca (M.S.); Cristoforo.Silvestri@criucpq.ulaval.ca (C.S.); Michel.Laviolette@criucpq.ulaval.ca (M.L.); lpboulet@med.ulaval.ca (L.-P.B.); vincenzo.dimarzo@criucpq.ulaval.ca (V.D.); 2 Canada Excellence Research Chair on the Microbiome-Endocannabinoidome Axis in Metabolic Health (CERC-MEND), Université Laval, Québec City, QC G1V 0A6, Canada; 3 Endocannabinoid Research Group, Institute of Biomolecular Chemistry, Consiglio Nazionale Delle Ricerche (CNR), 80078 Pozzuoli, Italy; m.kostrzewka@gmail.com (M.K.); rmvitale@icb.cnr.it (R.M.V.); aligresti@icb.cnr.it (A.L.); 4 Institut sur la Nutrition et les Aliments Fonctionnels, Centre NUTRISS, École de Nutrition, Faculté des Sciences de L’agriculture et de L’alimentation, Université Laval, Québec City, QC G1V 0A6, Canada; pier-luc.plante.1@ulaval.ca; 5 Faculté de Pharmacie de l’Université Laval and Centre de Recherche en Organogénèse Expérimentale de l’Université Laval/LOEX, Axe Médecine Régénératrice, Centre de Recherche du CHU de Québec-Université Laval, Québec City, QC G1V 0A6, Canada; Roxane.Pouliot@pha.ulaval.ca; 6 Joint International Unit between the Consiglio Nazionale delle Ricerche (CNR), 80078 Pozzuoli, Italy; 7 Canada on Chemical and Biomolecular Research on the Microbiome and Its Impact on Metabolic Health and Nutrition (UMI-MicroMeNu), Université Laval, Québec City, QC G1V 0A6, Canada

**Keywords:** endocannabinoid, linoleic acid, linoleoyl-glycerol, 13-HODE, 2-arachidonoyl-glycerol, anandamide, eicosanoid, eosinophils, neutrophils, *N*-linoleoyl-ethanolamine

## Abstract

The endocannabinoids 2-arachidonoyl-glycerol and *N*-arachidonoyl-ethanolamine are lipids regulating many physiological processes, notably inflammation. Endocannabinoid hydrolysis inhibitors are now being investigated as potential anti-inflammatory agents. In addition to 2-arachidonoyl-glycerol and *N*-arachidonoyl-ethanolamine, the endocannabinoidome also includes other monoacylglycerols and *N*-acyl-ethanolamines such as 1-linoleoyl-glycerol (1-LG) and *N*-linoleoyl-ethanolamine (LEA). By increasing monoacylglycerols and/or *N*-acyl-ethanolamine levels, endocannabinoid hydrolysis inhibitors will likely increase the levels of their metabolites. Herein, we investigated whether 1-LG and LEA were substrates for the 15-lipoxygenase pathway, given that both possess a 1*Z*,4*Z*-pentadiene motif, near their omega end. We thus assessed how human eosinophils and neutrophils biosynthesized the 15-lipoxygenase metabolites of 1-LG and LEA. Linoleic acid (LA), a well-documented substrate of 15-lipoxygenases, was used as positive control. *N*-13-hydroxy-octodecadienoyl-ethanolamine (13-HODE-EA) and 13-hydroxy-octodecadienoyl-glycerol (13-HODE-G)**,** the 15-lipoxygenase metabolites of LEA and 1-LG, were synthesized using Novozym 435 and soybean lipoxygenase. Eosinophils, which express the 15-lipoxygenase-1, metabolized LA, 1-LG, and LEA into their 13-hydroxy derivatives. This was almost complete after five minutes. Substrate preference of eosinophils was LA > LEA > 1-LG in presence of 13-HODE-G hydrolysis inhibition with methyl-arachidonoyl-fluorophosphonate. Human neutrophils also metabolized LA, 1-LG, and LEA into their 13-hydroxy derivatives. This was maximal after 15–30 s. Substrate preference was LA ≫ 1-LG > LEA. Importantly, 13-HODE-G was found in humans and mouse tissue samples. In conclusion, our data show that human eosinophils and neutrophils metabolize 1-LG and LEA into the novel endogenous 15-lipoxygenase metabolites 13-HODE-G and 13-HODE-EA. The full biological importance of 13-HODE-G and 13-HODE-EA remains to be explored.

## 1. Introduction

The endocannabinoids 2-arachidonoyl-glycerol (2-AG) and *N*-arachidonoyl-ethanolamine (AEA) are bioactive lipids mediating their effects mostly by activating the cannabinoid receptors CB_1_ and CB_2_. As such, they modulate several physiological responses, the most recognized ones being nociception, appetite, adipogenesis, and inflammation [1,2]. The levels of 2-AG and AEA are tightly regulated. As such, 2-AG is hydrolyzed into arachidonic acid (AA) by at least six lipases, notably the MAG lipase [3,4,5,6,7,8], while AEA is hydrolyzed into AA by the fatty acid amide hydrolases (FAAH)-1 and -2 and the *N*-acylethanolamine acid amidase (NAAA) [9,10,11]. Once hydrolyzed into AA, notably by leukocytes, 2-AG can be further metabolized into eicosanoids such as prostaglandins and leukotrienes [12,13,14]. Apart from being hydrolyzed into AA, 2-AG and AEA can also be directly metabolized by eicosanoid biosynthetic enzymes, the most documented metabolites being the cyclooxygenase-2-derived prostaglandins-glycerols (PG-G) and prostamides, respectively [15], as well as by CYP450 enzymes [16]. The 12- and the 15-lipoxygenase (LO) pathways were also shown to metabolize AEA and 2-AG into their 12-hydroxy and 15-hydroxy derivatives [17,18,19,20].

The 15-LO pathway is particularly relevant when investigating 2-AG, AEA, and their congeners from the monoacylglycerol and *N*-acyl-ethanolamine families. Indeed, human 15-LO-1 and 15-LO-2 metabolize numerous unsaturated fatty acids possessing a 1*Z*,4*Z*-pentadiene motif [20] and recent data indicate that additional *N*-acyl-ethanolamines are also metabolized by human 15-LOs. Indeed, LEA is notably metabolized into *N*-13-hydroxy-octoceaenoyl-ethanolamine (13-HODE-EA) by human recombinant 15-LO-1 and -2 [21], while *N*-docosahexaenoyl-ethanolamine (DHEA) is metabolized into 17-hydroxy-DHEA, 4,17-dihydroxy-DHEA, and 10,17-dihydroxy-DHEA by human neutrophils [22]. 

**Figure 1:**
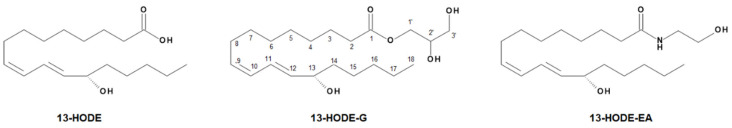
Chemical structures of 13-HODE, 13-HODE-G, and 13-HODE-EA.

Human eosinophils and neutrophils express 15-LOs, eosinophils mainly expressing the 15-LO-1, and neutrophils mainly expressing the 15-LO-2 and/or possibly another LO [20]. As such, both leukocyte types biosynthesize 13(S)-hydroxy-octodecadienoic acid (13-HODE) from linoleic acid (LA) [20,23,24]. Moreover, human recombinant 15-LO-1 and 15-LO-2 can biosynthesize 13-HODE-EA from *N*-linoleoyl-ethanolamine (LEA) [21]. We thus postulated that 1-linoleoyl-glycerol (1-LG) would be metabolized into 13-hydroxy-octodecadienoyl-glycerol (13-HODE-G) and that eosinophils and neutrophils would biosynthesize 13-HODE-G and 13-HODE-EA in response to 1-LG and LEA, respectively. The chemical structures of 13-HODE, 13-HODE-G, and 13-HODE-EA are shown in Figure 1.

Herein, we show that (1) the synthesis of 13-HODE-G was obtained from 1-LG using a strategy similar to what we recently reported for 13-HODE-EA [21]; (2) human recombinant 15-LO-1 and -2 biosynthesized 13-HODE-G and 13-HODE-EA from 1-LG and LEA, respectively; (3) human eosinophils and neutrophils metabolize 1-LG and LEA into 13-HODE-G and 13-HODE-EA, respectively; (4) serine hydrolase inhibition with MAFP is required to evaluate the ability of leukocytes to biosynthesize 13-HODE-G and (5) 13-HODE-G can be detected in several human and mouse tissues but, is unable to activate the same receptors as 1-LG.

## 2. Materials and Methods

### 2.1. Materials 

DMSO, Dextran, LC-MS grade MeOH, acetic acid, MeCN, water, and chloroform were purchased from Thermo Fisher Scientific (Ottawa, ON, Canada). HPLC water was purified with a Milli-Q system (Millipore, Oakville, ON, Canada). Lymphocyte separation medium was from Corning (Corning, NY, USA) and HBSS from Wisent Bioproducts (St-Bruno, QC, Canada). Methyl-arachidonoyl-fluorophosphonate (MAFP), linoleic acid, linoleoyl-glycerol, and linoleoyl-ethanolamine were purchased from Cayman Chemicals (Ann Arbor, MI, USA). Soybean lipoxygenase Type I-B, deuterated solvents, and all the reagents used for synthesis were purchased from Sigma Aldrich (Oakville, ON, Canada) unless otherwise specified and used without further purification. Anti-CD16 conjugated with MicroBeads and MACS columns were purchased from Miltenyi Biotec (Auburn, CA, USA). 

### 2.2. Synthesis and Purification of 1-LG-d_5_

For the synthesis of 1-LG-d_5_, we followed a previously documented strategy involving the enzyme Novozym 435 [25]. In brief, LA and glycerol-d_5_ (0.2 and 0.6 mmol, respectively) were dissolved in tert-butanol (1.2 mL). Then, molecular sieves and Novozym 435 (24 mg, 40% *w*/*w*) were added to the mixture. The reaction was initiated by placing the mixture in a thermoconstant orbital shaker (New Brunswick Scientific™, Edison, NJ, USA) and shook at 200 rpm (5 h, 60 °C). This led to the synthesis of 0.18 mmol of 1-LG-d_5_. After cooling at room temperature, removal of the solvents was performed under reduced pressure. Purification of 1-LG-d_5_ was achieved by silica gel chromatography using CHCl_3_-MeOH (98:2, *v*/*v*). 

### 2.3. Synthesis and Purification of 13-HODE-G and 13-HODE-G-d_5_

Our strategy to synthesize 13-HOGE-G (and 13-HODE-G-d_5_) was elaborated based on the fact that soybean lipoxygenase can transform *N*-linoleoyl-ethanolamine (LEA) into 13-HODE-EA [26], a process we could repeat [21]. In brief, 0.01 mmol of 1-LG or 1-LG-d_5_ was diluted in 100 mL of Na_2_B_4_O_7_ (50 mM, pH 12). The reaction was initiated by adding 20 mg of soybean lipoxygenase (0.2 mg/1 mL) and stirred for 15 min at room temperature. The hydroperoxides were then reduced by adding 2 mL of a freshly prepared sodium borohydride solution (1 M in ethanol). The solution was acidified by the dropwise addition of glacial acetic acid (500 µL over 1 min) and gently stirred 15 min until foaming stopped. The resulting reaction products were then extracted with 300 mL CHCl_3_, dried by adding Na_2_SO_4_, filtered, and concentrated *in vacuo*. The resulting residue was filtered over glass wool and purified by RP-HPLC as previously described [21] and showed a retention time of 9.0 min. The solvent was evaporated under vacuum and the purified product was weighted.

### 2.4. Characterization with 1D and 2D NMR 

All products and synthetic intermediate structures were assigned using ^1^H, ^13^C, COSY, HSQC NMR spectra, and were recorded in 5 mm tubes on an Agilent 500 MHz DD2 system (Agilent NMR System, Santa Clara, CA, USA) with a OneNMR probe. ^1^H NMR and ^13^C NMR chemical shifts are referenced to residual protons in deuterated solvents. Multiplicities are reported as singlet (s), doublet (d), triplet (t), quintet (q), multiplet (m), broad (br), or overlapping (ov). Chemical shifts are reported in ppm. Coupling constants are reported in Hz. Protons were assigned using integrated ^1^H-NMR and 2D ^1^H, ^1^H-gCOSY spectra to detect correlation between neighboring protons. The quaternary carbon was determined using the 1D ^13^C experiments. Protons were then assigned to carbons using 2D ^1^H, ^13^C-HSQC spectra showing single through-bond relationships and CH were discriminated from CH_2_ and CH_3_ with crosspeaks of different colors.

### 2.5. Characterization with HR-ESI-MS/MS Spectrometry

High-resolution mass spectrometry was performed on a Thermo Orbitrap Fusion tribrid mass spectrometer (Thermo Scientific, Bremen, Germany) using a Chemyx F100T2 syringe pump (Chemyx Inc., Stafford, TX, USA) running at 5 uL/min. The mass spectrometer was operated in positive or negative ionization modes using the orbitrap detector at a resolution of 60,000. For CID, the collisional energy was set between 0 and 50 eV.

### 2.6. Enzymatic Assays

Recombinant human 15-LO-1 and 15-LO-2 enzymes were added to HEPES 25 mM + 0.01% Triton (14 μg/mL for 15-LO-1, 56 μg/mL for 15-LO-2). A total of 10 μM of the fatty acids were added to the buffer for 5 min (15-LO-1) or 15 min (15-LO-2), at 37 °C. The final volume of the reaction mix was 100 µL. After the incubation, 50 µL of NaBH_4_ 1M was added to the reaction, for 5 min at 37 °C. The reactions were stopped by adding 0.5 mL of cold (−20 °C) methanol +0.01% acetic acid and kept at −30 °C until further processing.

### 2.7. Analysis of 13-HODE-G and 13-HODE-EA by LC-MS/MS

For tissues samples, ~10 mg of tissue was put in 500 µL of Tris Buffer, crushed with a pestle, mixed with 500 µL of cold (−20 °C) MeOH containing the internal standards, centrifuged at 10,000× *g* and the supernatant collected. All Samples were processed by adjusting the final volume to 1 mL (incubation buffer-MeOH mixture, 1/1, *v*/*v* plus the internal standards). Samples (1 mL) were acidified with 0.575% of acetic acid and mixed with 1 mL CHCl_3_. Samples were then vortexed for 1 min, then centrifuged at 3000× *g* for 5 min. The organic phase (lower phase) was harvested, dried under a stream of nitrogen and reconstituted with 25 μL of solvent A (H_2_O containing 1 mM of ammonium hydroxide and 0.05% of acetic acid) and 25 μL of solvent B (MeCN/H_2_O, 95/5, *v*/*v* containing 1 mM of ammonium hydroxide and 0.05% of acetic acid). A total of 40 μL of the samples were injected onto an HPLC column (Kinetex C8, 150 × 2.1 mm, 2.6 μm, Phenomenex, Torrance, CA, USA) and eluted at a flow rate of 0.4 mL/minute using a discontinuous gradient as described previously [27]. The HPLC system was interfaced with the electrospray source of a Shimadzu 8050 triple quadrupole mass spectrometer and mass spectrometric analysis was done in the negative or positive ion mode using multiple reaction monitoring for the specific mass transition of the metabolites (Table 1). 15-LO metabolites were quantified using calibration curves, as described before [20]. 

### 2.8. Isolation of Human Neutrophils and Eosinophils

Neutrophils and eosinophils were isolated from the peripheral blood of healthy volunteers as described before [28]. First, platelet-rich plasma was discarded by centrifugation and erythrocytes were sedimented using HBSS containing 6% of dextran. Then, granulocytes were separated from mononuclear cells using a discontinuous gradient with the Lymphocyte separation medium. Residual erythrocytes were removed by hypotonic lysis with sterile water. Eosinophils and neutrophils were separated using magnetic bead conjugated anti-CD16 and MACS column, according to the manufacturer’s instructions. Purity and viability were >95% and were assessed by Diff-Quick coloration and Trypan blue exclusion, respectively. 

### 2.9. Cell Stimulations

Prewarmed (37 °C) suspensions of human neutrophils (5 × 10^6^ cells/mL) or eosinophils (10^6^ cells/mL) in HBSS containing 1.6 mM CaCl_2_ were incubated with 1 µM MAFP for 5 min before the addition of LA, 1-LG, or LEA for different times or concentrations (see legends of Figures 3–8). Adenosine deaminase (0.3 U/mL) was added to neutrophil suspensions 10 min before the addition of the stimuli in order to remove the inhibitory constraint that adenosine has on their numerous responses [29]. Incubations were stopped by adding one volume of cold (−20 °C) MeOH containing the internal standards (Table 1). Samples were then kept at −30 °C until further processing. Lipids were extracted and quantified by LC-MS/MS as described above. 

### 2.10. CB_1_ and CB_2_ Binding Assays

The analysis of CB_1_ and CB_2_ receptor binding was performed exactly as described in [21].

### 2.11. Analysis of PPAR-α, PPAR-γ and TRPV1 Functional Activity

These assays we preformed exactly as described in [21]. 

### 2.12. Computational Analysis of PPAR-α Binding

13-HODE was retrieved from Pubchem (CID: 5282947). 13-HODE was then fully optimized using the GAMESS program [30] at the Hartree–Fock level with the STO-3G basis set and subjected to HF/6-31G*/STO-3G single-point calculations to derive the partial atomic charges using the RESP procedure [31]. Docking studies were performed with AutoDock v4.2 [32] by using the PPAR-α crystallographic structure (PDB ID: 2P54). Protein and ligands were processed with AutoDock Tools (ADT) package version 1.5.6rc1 [32] to merge non-polar hydrogens and calculate Gasteiger charges. Grids for docking evaluation with a spacing of 0.375 Å and 60 × 60 × 60 points, centered on the ligand binding site, were generated using the program AutoGrid v4.2 included in Auto-dock v4.2 distribution. A total of 100 molecular docking runs for each docking calculation were performed adopting a Lamarckian Genetic Algorithm (LGA) and the protocol already published [33]. Flexibility was used for all rotatable bonds of the docked ligands. For each docking run, the representative poses were selected on the basis of best binding energy values and visual inspection and underwent energy minimization with Amber16 package [34] using ff14SB version of AMBER ff14SB force field for the protein and gaff parameters for the ligand. To perform molecular dynamics simulations in solvent, the complexes were confined in TIP3P water periodic truncated octahedron boxes exhibiting a minimum distance between solute atoms and box surfaces of 10 Å, using the leap module of the AmberTools16 package. Molecular dynamics simulations were performed using a protocol published elsewhere [33]. Production runs were carried out for 100 ns. The cpptraj module of AmberTools16 and program UCSF Chimera v1.10.1 were used to perform molecular dynamics analysis and to draw the figures, respectively.

### 2.13. Ethics Committee Approval

This study was approved by the local ethics committee (Comité d’éthique de la recherche de l’Institut universitaire de cardiologie et de pneumologie de Québec-Université Laval) and all participants signed an informed consent form.

## 3. Results

### 3.1. Synthesis of 1-LG-d_5_, 13-HODE-G, and 13-HODE-d_5_

**Figure 2:**
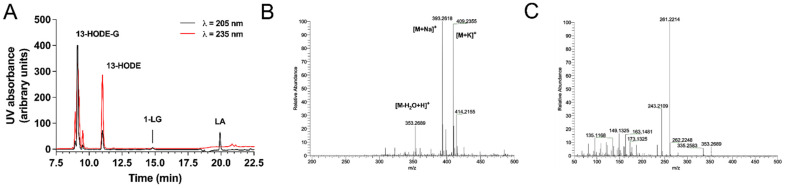
RP-HPLC chromatogram and mass spectrometry analysis of 13-HODE-G. (**A**) Separation of 13-HODE-G from other possible contaminants. The reaction products (~350 µg) of 1-LG with soybean lipoxygenase were loaded onto the HPLC column and eluted as described in Material and Methods. (**B**) Positive electrospray ionization mass spectrometry (ESI+) of 13-HODE-G. Positive electrospray ionization mass spectrometry (ESI+) of 13-HODE-G yielded three main cations; [M-H_2_O+H]^+^ was found at *m*/*z* 353.2689, [M+Na]^+^ at *m*/*z* 393.2618 and [M+K]^+^ at *m*/*z* 409.2355. (**C**) Collision-Induced Dissociation (CID) at Higher-energy collisional dissociation (HCD) of 20 eV of 13-HODE-G. CID of [M-H_2_O+H]^+^ at *m*/*z* 353.2689 led to the dominant product ion of *m*/*z* 261.2214.

We recently developed a strategy to synthesize 13-HODE-EA and 13-HODE-EA-d_4_ from LEA and LEA-d_4_, respectively [21]. We basically applied a similar strategy to synthesize 1-LG-d_5_, 13-HODE-G-d_5,_ and 13-HODE-G (Figure S1). First, we synthesized 1-LG-d_5_ with Novozyme435, given that this compound was not commercially available. This reaction was very efficient (~90% yield), and we confirmed the molecular structure of the latter using ^1^H and ^13^C NMR (Figures S2 and S3). We next performed the lipoxygenation of 1-LG and 1-LG-d_5_ using soybean lipoxygenase (Figure S1). The yield of this reaction was 30 and 40% for 13-HODE-G and 13-HODE-d_5_, respectively. 13-HODE-G (and 13-HODE-d_5_) was easily separated from 13-HODE by RP-HPLC and led to increased UV absorbance at 235 nm, indicating the formation of a conjugated diene. (Figure 2A). Of note, the elution peak of 13-HODE-G consisted of a mixture of the *sn*-2 and -1(3) isomers. 

The structure of the conjugated diene of 13-HODE-G and 13-HODE-G-d_5_ were confirmed by NMR as *cis-trans* on the basis of the coupling constants at positions 9–12. Indeed, the value of J_9–10_ was 11.0 Hz, indicating the presence of a *cis* configuration double bond, whereas the coupling constant of J_11–12_ was 15.2 Hz, characteristic of *trans* geometry (Table 2, Figures S4, S5, S8 and S9).

In contrast to 1-LG-d_5_, for which the signal of H13 was visible, the same signal in the ^1^H-NMR spectra of 13-HODE-G was completely overlapped by the 2′-CH2 signal of the glycerol. Therefore, HSQC and COSY experiments were set up to elucidate the presence of the hydroxyl group bound to the C13. The HSQC data showed a direct correlation C13-H13 (δH 4.16 ppm, δC 72.9 ppm) and the value of the δC was typical of a carbon directly linked to hydroxyl group for the lipoxygenase metabolites (Figures S6 and S10). The COSY data showed crosspicks between H13-H14 (δH-13 4.20, δH-14 1.25) and H13-H12 (δH-13 4.20, δH-12 5.67), which led to define the exact position of the hydroxyl group on the C13 (Figures S7 and S11). 

We next performed a high-resolution scan of 13-HODE-G. Positive electrospray ionization mass spectrometry (ESI+) of 13-HODE-G yielded three main cations, i.e., the protonated molecule with loss of water [M-H_2_O+H]^+^ at 353.2689, as well as sodium adduct [M+Na]^+^ at *m*/*z* 393.2618 and a potassium adduct [M+K]^+^ with K^+^ at *m*/*z* 409.2355 (Figure 2B). The collision-induced decomposition (CID) of the [M-H_2_O+H]^+^ ion at *m*/*z* 353.2689 yielded the predominant product ion at *m*/*z* 261.2213 ([13-HODE-G-H_2_O-glycerol]^+^), which is produced by loss of the hydroxyl group at C13 combined with the neutral loss of the glycerol (Figure 2C), as described for 2-AG [35].

### 3.2. Biosynthesis of 13-HODE-G by Human Recombinant 15-LO-1 and 15-LO-2

**Figure 3:**
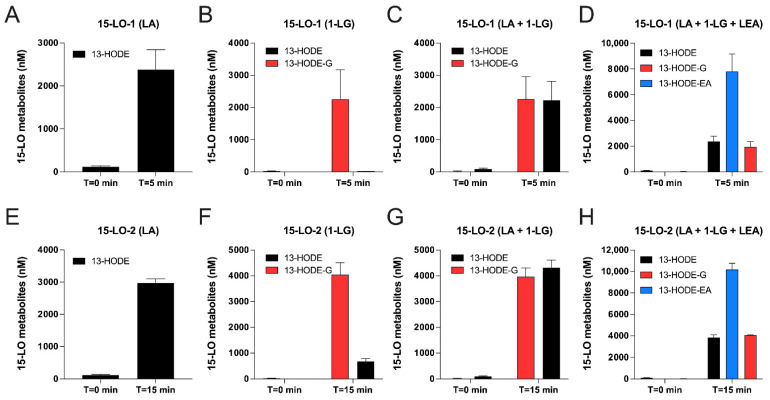
Metabolism of 1-LG, LA and LEA by human recombinant 15-LO-1 and 15-LO-2. Human recombinant 15-LO-1 (**A**–**D**) or 15-LO-2 (**E**–**H**) were incubated at 37 °C in HEPES 25 mM containing 0.01% triton. A total of 10 μM of LA (**A**,**D**,**E**,**H**); 1-LG (**B**,**D**,**F**,**H**) or LEA (**D**,**H**) were added for 5 min (15-LO-1) or 15 min (15-LO-2). NaBH_4_ was then added for 5 min and incubations were stopped by the addition of 0.5 mL of cold (−20 °C) MeOH containing 0.01% acetic acid and the internal standards. Samples then were processed for LC-MS/MS analyses as described in methods. Results are the mean (±SEM) of 3 independent experiments.

We next assessed whether human recombinant 15-LO-1 and 15-LO-2 could biosynthesize 13-HODE-G from 1-LG, given that 2-LG is rapidly isomerized into 1-LG in the presence of water and that the experiments were done in aqueous buffers. In these experiments, the LA-induced 13-HODE synthesis was used as positive control (Figure 3A,E).

Both 15-LO-1 and 15-LO-2 biosynthesized 13-HODE-G from 1-LG (Figure 3B,F). At 10 µM 1-LG, the efficacy of the reaction was 23% for 15-LO-1 and 40% for 15-LO-2. Of note, the 13-HODE-G:13-HODE ratio was 1:1 for both enzymes, indicating that they metabolized 1-LG and LA to comparable levels. (Figure 3C,G). Given that both enzymes can also biosynthesize 13-HODE-EA from LEA [21], we also tested the impact of a combination of LA, 1-LG, and LEA on the biosynthesis of 13-HODE, 13-HODE-G, and 13-HODE-EA, respectively. The combination of LA, 1-LG, and LEA led to the biosynthesis of 13-HODE-EA > 13-HODE-G = 13-HODE for both 15-LO-1 and 15-LO-2 (Figure 3D,H). In these experiments, the incubation of 1-LG and/or LEA in absence of enzyme did not lead to the production of 13-HODE-G and/or 13-HODE-EA.

### 3.3. Human Eosinophils Metabolize LA, 1-LG, and LEA via the 15-LO Pathway

**Figure 4:**
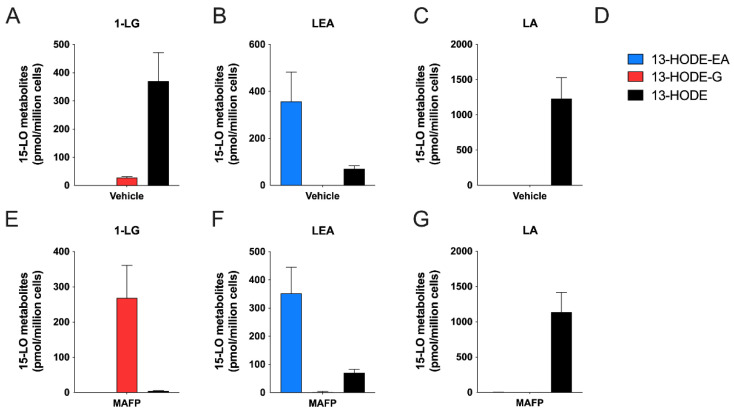
Metabolism of LA, 1-LG or LEA by human eosinophils. Pre-warmed human eosinophil suspensions (37 °C, 10^6^ cells/mL in HBSS containing 1.6 mM CaCl_2_) were treated with DMSO (**A**–**C**) or MAFP 1 μM (**E**–**G**) for 5 min then stimulated with 3 μM of (**A**,**E**) 1-LG, (**B**,**F**) LEA, (**C**,**G**) LA for another 5 min. (**D**) Metabolite legend for panels. (**A**–**G**) Incubations were stopped by the addition of one volume of cold (−20 °C) MeOH containing the internal standards. Samples then were processed for LC-MS analysis as described in methods. Results are the mean (±SEM) of 4–5 independent experiments.

Given the ability of human 15-LO enzymes to biosynthesize 13-HODE-EA and 13-HODE-G from LEA and 1-LG, respectively ([21] and Figure 3), we next investigated if human eosinophils and neutrophils, which are recognized to biosynthesize 13-HODE in response to LA [20,23,24], could also biosynthesize 13-HODE-EA and 13-HODE-G in response to their respective precursors. This possibility was very plausible, given that human eosinophils and neutrophils can metabolize the endocannabinoids 2-AG and AEA into their 15-hydroxylated derivatives [18,20]. To this end, we first treated human eosinophils with 3 µM 1-LG, LEA or LA for 5 min (Figure 4). Human eosinophils metabolized 1-LG into 13-HODE-G and 13-HODE, with a predominance of 13-HODE (Figure 4A). This was expected as we previously reported that both 2-AG and 15-HETE-G were respectively hydrolyzed by human eosinophils into AA and 15-HETE, a phenomenon that was prevented by MAFP [36]. Furthermore, 1-LG and 13-HODE-G were also hydrolyzed by eosinophils, a phenomenon partially prevented by MAFP (data not shown). We thus repeated the experiment in the presence of MAFP, to prevent the hydrolysis of both 1-LG and 13-HODE-G. In agreement with the strong increase in 2-AG and 15-HETE-G half-life when their hydrolysis is inhibited [36], MAFP enhanced the 1-LG-induced biosynthesis of 13-HODE-G tenfold (Figure 4D), without affecting the biosynthesis of 13-HODE-EA induced by LEA and of 13-HODE induced by LA (Figure 4E,F). Consequently, and to better assess the biosynthesis of 13-HODE-G, all further experiments involving 1-LG were performed in presence of MAFP. Human eosinophils also biosynthesized 13-HODE-EA and 13-HODE in response to LEA (Figure 4B,E). As expected, human eosinophils metabolized LA into 13-HODE (Figure 4C,F). Of note, human eosinophils biosynthesized more 13-HODE than 13-HODE-G or 13-HODE-EA, suggesting a better uptake of LA or a preferential metabolism of LA by eosinophils vs. exogenous 1-LG or LEA.

**Figure 5:**
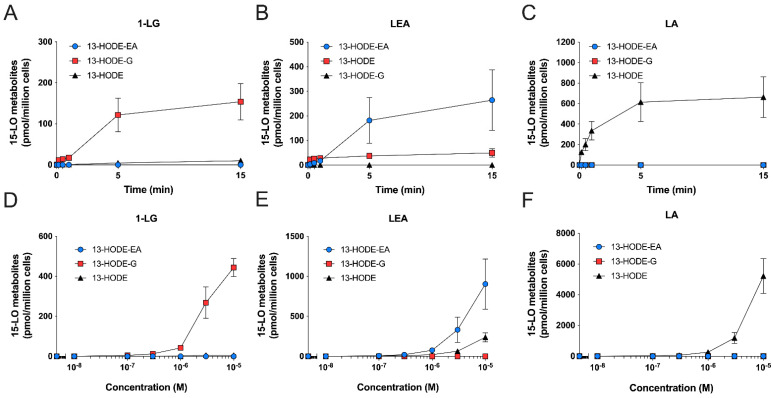
Time- and concentration-dependent synthesis of 15-LO metabolites from 1-LG, LEA, and LA by human eosinophils. Pre-warmed human eosinophils suspensions (10^6^ cells/mL in HBSS containing 1.6 mM CaCl_2_) were treated with 3 μM of (**A**) 1-LG, (**B**) LEA, or (**C**) LA for the indicated times. (**D**–**F**) Eosinophils were treated with increasing concentration of (**D**) 1-LG, (**E**) LEA or (**F**) LA for 5 min. In experiments involving 1-LG, cells were pretreated with MAFP for 5 min prior to the addition of 1-LG. Incubations were stopped by adding one volume of cold (−20 °C) MeOH containing the internal standards. Samples then were processed for LC-MS/MS analyses as described in methods. Data are the mean (±SEM) of 4–5 independent experiments.

We next performed kinetic experiments to better establish the biosynthetic profiles of 13-HODE-G and 13-HODE-EA by human eosinophils, compared to that of 13-HODE. 1-LG, LEA, and LA at 3 µM stimulated the biosynthesis of their 15-lipoxygenase metabolite in a time-dependent manner with maximal levels obtained after 15 min, which was the longest time point studied (Figure 5A–C). Of note, there was a small delay of ~1 min before 1-LG and LEA were metabolized into their 15-lipoxygenase counterparts. This was not observed with the LA-induced 13-HODE biosynthesis. Concentration-response experiments showed that the biosynthesis of 13-HODE, 13-HODE-G, and 13-HODE-EA was concentration-dependent (Figure 5D–F). The biosynthesis of these metabolites could be detected with 100 nM of substrate and was maximal at 10 µM, which was the highest concentration studied. Of note, neither 13-HODE, 13-HODE-G, nor 13-HODE-EA were detected in vehicle-treated eosinophils.

### 3.4. Human Neutrophils Also Metabolize LA, 1-LG, and LEA into 13-Hydroxylated Compounds

**Figure 6:**
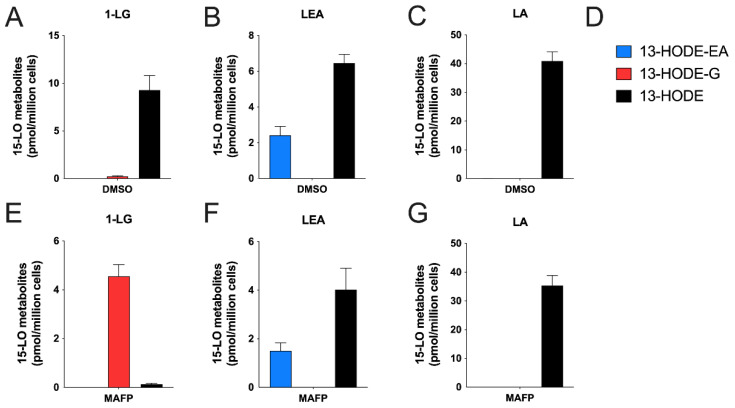
15-LO metabolite production in response to LA, 1-LG, or LEA by human neutrophils. Pre-warmed human neutrophil suspensions (37 °C, 5 × 10^6^ cells/mL in HBSS containing 1.6 mM CaCl_2_) were pre-treated with DMSO (**A**–**C**) or MAFP (**E**–**G**) for 5 min before the addition of 3 μM of either (**A**,**E**) 1-LG, (**B**,**F**) LEA, (**C**,**G**) LA. (**D**) Metabolite legend for panels **A**–**G**. Incubations were stopped after 5 min by the addition of one volume of cold (−20 °C) MeOH containing the internal standards. Samples then were processed for LC-MS/MS analyses as described in methods. Results are the mean (±SEM) of 4 independent experiments.

Human neutrophils are also capable of metabolizing fatty acids and endocannabinoids into their 15-LO derivatives, possibly via the 15-LO-2 and/or another unidentified enzyme [20]. We therefore tested if they would also metabolize 1-LG and LEA into their 13-HODE derivatives. Human neutrophils biosynthesized 13-HODE-G, 13-HODE-EA or 13-HODE in response to 3 µM 1-LG, LEA or LA respectively (Figure 6A–C). In contrast to eosinophils, which produced high nM levels of the 15-lipoxygenase metabolites (Figure 4 and Figure 5), neutrophils biosynthesized low nM levels of the investigated 15-LO metabolites. Moreover, and in agreement with their strong ability to hydrolyze 2-AG and 15-HETE-G in a MAFP-sensitive manner [36], 1-LG-treated neutrophils biosynthesized 20 times more 13-HODE-G and 65 times less 13-HODE in the presence of MAFP (Figure 6D). As for eosinophils, MAFP did not impact the ability of neutrophils to biosynthesize 13-HODE-EA and 13-HODE in response to LEA and LA, respectively (Figure 6E,F). While the treatment of human neutrophils with 3 µM LA led to the exclusive biosynthesis of 13-HODE, the main product detected with the incubation with LEA was 13-HODE (ratio 13-HODE /13-HODE-EA of 2.67 ± 0.590) and MAFP (which also inhibits the FAAH [37]) had no effect on this ratio. 13-HODE, 13-HODE-G, or 13-HODE-EA were not detected in vehicle-treated neutrophils. Furthermore, 1-LG and 13-HODE-G were also hydrolyzed by neutrophils, and this hydrolysis was prevented by MAFP (data not shown).

**Figure 7:**
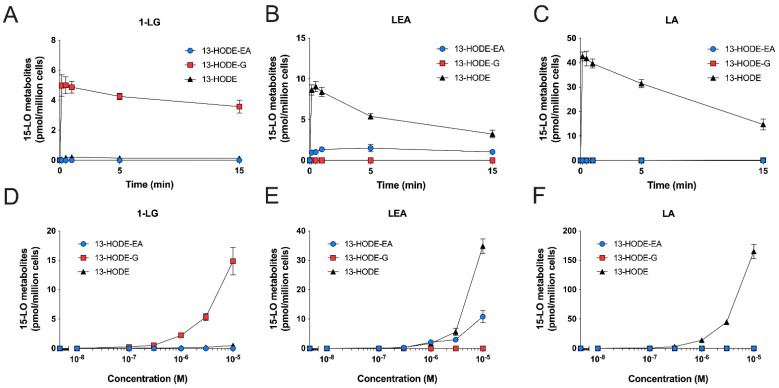
Time- and concentration-dependent biosynthesis of 15-LO metabolites from 1-LG, LEA, and LA by human neutrophils. Pre-warmed human neutrophils suspensions (37 °C, 5 × 10^6^ cells/mL in HBSS containing 1.6 mM CaCl_2_) were treated with 3 μM of (**A**) 1-LG, (**B**) LEA, or (**C**) LA for the indicated times. (**D**–**F**) Neutrophils were treated with increasing concentration of (**D**) 1-LG, (**E**) LEA, or (**F**) LA for 5 min. In experiments involving 1-LG, cells were pretreated with 1 µM MAFP for 5 min prior to the addition of 1-LG. Incubations were stopped by the addition of one volume of cold (−20 °C) MeOH containing the internal standards. Samples then were processed for LC-MS/MS analyses as described in methods. Data are the mean (±SEM) of 4 independent experiments.

We next performed kinetic and concentration-response experiments with human neutrophils to further characterize their ability to biosynthesize 13-HODE-G and 13-HODE-EA. The appearance of 13-HODE, 13-HODE-G, and 13-HODE-EA was much faster in neutrophils than in eosinophils, in line with our previous data using other 15-LO substrates [20]. Indeed, human neutrophils rapidly (10–30 s) biosynthesized 13-HODE derivatives upon their incubation with 3 µM LA, 1-LG, or LEA (Figure 7). For the biosynthesis of 13-HODE-G and 13-HODE-EA, the levels remained relatively stable from 0.5 to 15 min (Figure 7A,B). In contrast, and as previously reported [20], the biosynthesis of 13-HODE in response to LA peaked at 10 s, then slowly declined until 15 min (Figure 7C). Concentration-response experiments showed that the biosynthesis of 13-HODE, 13-HODE-G, and 13-HOGE-EA was also concentration-dependent in neutrophils (Figure 7D–F). The biosynthesis of these metabolites could be detected with 300 nM of substrate and was maximal at 10 µM, which was the highest concentration investigated. 

### 3.5. Substrate Preference of Eosinophils and Neutrophils

**Figure 8:**
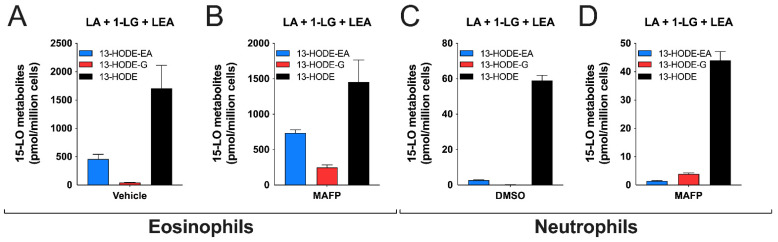
Substrate preference by human eosinophils and neutrophils. Pre-warmed (37 °C) human eosinophil suspensions (10^6^ cells/mL in HBSS containing 1.6 mM CaCl_2_) or neutrophil suspensions (5 × 10^6^ cells/mL in HBSS containing 1.6 mM CaCl_2_) were pre-treated with DMSO (**A**,**C**) or MAFP (**B**,**D**) for 5 min before the addition of a mixture of 1-LG, LEA, and LA at 3 μM each. Incubations were stopped after 5 min by the addition of one volume of cold (−20 °C) MeOH containing the internal standards. Samples then were processed for LC-MS/MS analyses as described in methods. Results are the mean (±SEM) of 4 independent experiments.

We recently showed that when investigating the biosynthesis of 15-LO metabolites by eosinophils and neutrophils, combining different substrates together does not drastically impact substrate preference vs. recombinant enzymes [20]. We thus performed another series of experiments in which we treated eosinophils and neutrophils from the same donors with a combination of LA, 1-LG, and LEA, at 3 µM each (Figure 8). Eosinophils were consistently better at converting the three substrates than neutrophils, with ~500 times more 13-HODE-EA, ~60 times more 13-HODE-G, and ~30 times more 13-HODE. Interestingly, the 13-HODE:13-HODE-EA:13-HODE-G ratio was 6:3:1 for MAFP-treated eosinophils and 30:1:3 for neutrophils, supporting the above finding that among the LA-containing lipids we tested, LA was most strongly metabolized by the two cell types.

### 3.6. Inhibition of 13-HODE-EA and 13-HODE-G Biosynthesis by BLX-3887 and NDGA

**Figure 9:**
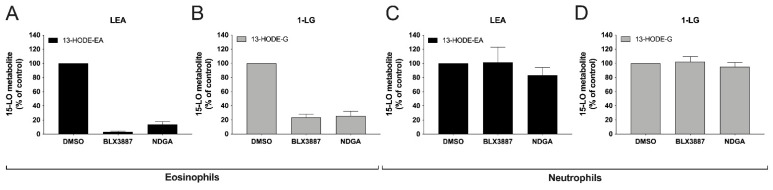
Inhibition of 13-HODE-G and 13-HODE-EA biosynthesis by 15-LO inhibitors in human eosinophils and neutrophils. Pre-warmed (37 °C) human (**A**,**B**) eosinophil suspensions (10^6^ cells/mL in HBSS containing 1.6 mM CaCl_2_) or (**C**,**D**) neutrophil suspensions (5 × 10^6^ cells/mL in HBSS containing 1.6 mM CaCl_2_) were pre-treated with DMSO, BLX-3887 (10 µM) or NDGA (10 µM) for 5 min before the addition of 3 μM (**A**,**C**) LEA or (**B**,**D**) 1-LG. Incubations were stopped 5 min after LEA or 1-LG treatment by the addition of one volume of cold (−20 °C) MeOH containing the internal standards. Samples then were processed for LC-MS/MS analyses as described in methods. Results are the mean (±SEM) of 4 independent experiments.

We next investigated if the biosynthesis of 13-HODE-EA and 13-HODE-G was the consequence of 15-LO-1 activity by performing a series of experiments in presence of the 15-LO-1-selective inhibitor BLX-3887 [38] or the LO inhibitor NDGA [39,40]. As expected, the treatment of eosinophils with BLX-3887 (10 µM) strongly inhibited the 1-LG-induced 13-HODE-G biosynthesis by ~75% and the LEA-induced 13-HODE-biosynthesis by ~95% (Figure 9A,B). In contrast, the incubation of neutrophils with BLX-3887 or NDGA did not inhibit the biosynthesis of the investigated 15-LO metabolites (Figure 9C,D). This was expected as we previously reported, as BLX-3887 and NDGA did not inhibit the biosynthesis of 15-LO metabolites from exogenously added fatty acids in neutrophils [20].

### 3.7. Detection of 13-HODE-G In Vivo

We previously documented that 13-HODE-EA was found in human saliva and skin samples [21]. While we could not detect 13-HODE-G in human saliva samples, we found this metabolite in human epidermis samples (21.9 ± 6.2 fmol/mg tissue, mean ± SEM, *n* = 5). Furthermore, 13-HODE-G was found in other tissues from mice, notably the gastrointestinal tract (Table 3), the liver and the adipose tissue [41], indicating that 13-HODE-G is also an endogenous mediator.

### 3.8. Binding Assays

**Figure 10:**
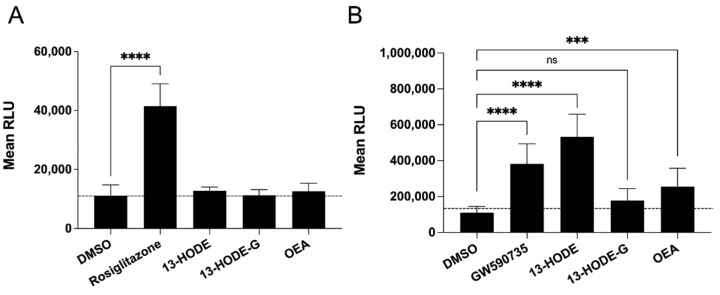
Impact of 13-HODE and 13-HODE-G on PPAR activity. Cells were incubated with 30 µM 13-HODE, 13-HODE-G, or OEA overnight and PPAR activity was measured using the PPAR assay kits according to the manufacturers’ instructions. Rosiglitazone (10 µM) and GW 590735 (100 nM) were utilized as positive controls for the (**A**) PPAR-γ and (**B**) PPAR-α assays, respectively. Data represent the mean (± SEM) of three (PPAR-γ) or five (PPAR-α) experiments, each performed in triplicates. *p* values were obtained by performing a 2-way ANOVA with a Sidak multiple comparison tests. **** *p* < 0.0001, *** *p* < 0.001. ns: *p* > 0.05.

We next sought to determine whether 13-HODE-G could bind/activate receptors belonging to the endocannabinoidome, notably the CB_1_, CB_2_, and TRPV_1_, as well as PPAR-α and -γ. Given that 13-HODE-G appears to be rapidly hydrolyzed into 13-HODE (Figure 4, Figure 6 and Figure 8), we also tested the impact of 13-HODE in our different assays. To this end, we first performed binding assays for the CB_1_ and CB_2_ receptors. While 1-AG was bound to both receptors, 1-LG was only bound to the CB_1_ receptor. In contrast, neither 13-HODE nor 13-HODE-G exhibited any binding affinity for the CB_1_ and CB_2_ receptors up to 10 µM (Table 4). As for TRPV_1_, while 1-AG and 1-LG displayed functional activity toward this channel, neither 13-HODE-G nor 13-HODE activated that receptor again (Table 5). Finally, 13-HODE-G did not stimulate the transcriptional activity of PPAR-α or -γ, in contrast to robust effect of 13-HODE on PPAR-α (Figure 10B).

### 3.9. Theoretical Complex of PPARα with 13-HODE

**Figure 11:**
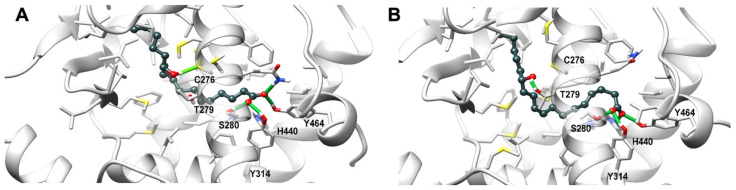
Representative frames from molecular dynamics of PPARα (light gray) in complex with 13-HODE (dark grey) shown in ball and stick representation. Panel (**A**,**B**) show the docking pose I and pose II of the ligand, respectively. Protein residues within 5 Å from the ligands are shown in stick representation. H-bonds are shown as green stick. Hydrogen, nitrogen, oxygen, and sulfur atom are painted white, blue, red, and yellow, respectively. A transparent surface for ribbons was used wherever they hide the ligand-binding site.

To get insight into the binding mode of 13-HODE into PPARα ligand binding domain (LBD) a molecular docking plus molecular dynamics simulations has been carried out. Two docking starting poses, i.e., pose I and pose II, sharing the orientation of the carboxylate group, which points toward the polar residues in the LBD, that is His440 (helix H10/11), Ser208 (Helix H3), and Tyr464 (helix H12), but differing in the conformation of the alkyl chain, were selected for the subsequent molecular dynamics simulations. The representative frames from molecular dynamics for each pose are shown in Figure 11. Both poses stably engages a network of H-bonds between the carboxylic group and His440 (helix H10/11), Ser208 (Helix H3), Tyr314 (helix H5), and Tyr464 (helix H12), a residue critical for a complete activation of PPAR-α, while the hydroxyl group forms a stable H-bond either with Cys276 or Thr279 (helix H3) sidechain in pose I and pose II, respectively (Figure 11). The alky chain is hosted in the hydrophobic pocket formed by the helix bundle and the β-sheet and wraps around helix H3 in a horseshoe conformation.

## 4. Discussion

Over the last forty years, several studies have been documenting that the 15-LO pathway participates in the biosynthesis of numerous oxylipins from various substrates. However, while it has been documented that 15-LO enzymes can metabolize the endocannabinoids 2-AG and AEA into 15-HETE-G and 15-HETE-EA, respectively [1,18,20,42], there is a knowledge gap regarding the biosynthesis of 15-LO-derived metabolites of other monoacylglycerols and *N*-acyl-ethanolamines, the lipid families to which 2-AG and AEA belong. In this regard, we recently documented the metabolism of the endocannabinoidome mediator LEA into 13-HODE-EA by human recombinant 15-LO-1 and 15-LO-2 [21]. In this study, we aimed at documenting if 1-LG could also be metabolized by the 15-LO pathway and if LEA and 1-LG could be metabolized by 15-LO-expressing leukocytes.

The data presented herein show that (1)1-LG and 1-LG-d_5_ can be synthesized using soybean LO into 13-HODE-G and 13-HODE-G-d_5_, respectively; (2) recombinant 15-LO-1 and 15-LO-2 have the ability to metabolize 1-LG into 13-HODE-G; (3) human eosinophils and neutrophils metabolize LEA into 13-HODE-EA and 1-LG into 13-HODE-G in a time- and concentration-dependent manner; (4) the biosynthesis of 13-HODE-G can only be correctly assessed in presence of the serine hydrolase inhibitor MAFP in both eosinophils and neutrophils; (5) in presence of an equal concentrations of substrates, the ratio of 13-HODE:13-HODE-EA:13-HODE-G biosynthesis was 6:3:1 in eosinophils and 30:3:1 in neutrophils; (6) the biosynthesis of the investigated 15-LO metabolites was blocked by the 15-LO-1 inhibitor BLX3887 in eosinophils but not in neutrophils; (7) 13-HODE-G is very weakly active or inactive at targets shown previously and here to be activated by its parent compound. 1-LG, i.e., CB_1_, CB_2_, TRPV_1_, and PPAR-α or -γ, whereas 13-HODE was found here to activate PPAR-α; and (8) 13-HODE-G was found in numerous tissues from mice and humans.

Odaneth and colleagues previously showed that stearoyl-glycerol synthesis was successfully achieved by performing an esterification using glycerol and stearic acid [25]. Herein, we adapted this strategy that was key for the synthesis of 1-LG-d_5_, the latter being commercially unavailable. This latter compound was then used here for the quantitative analysis of 1-LG by LC-MS/MS. The yield was very good (~90%) and 1-LG-d_5_ was easily separated from LA by RP-HPLC. In addition, we provide compelling evidence that soybean lipoxygenase allows the synthesis of 13-HODE-G and 13-HODE-G-d_5_ from 1-LG and 1-LG-d_5_, respectively. The yields of the reactions were lower, under the experimental conditions described, than what we reported for 13-HODE-EA (~45%; [21]). Indeed, the reaction of 1-LG with soybean lipoxygenase also led to the production of 13-HODE and LA, indicating that the basic conditions in which the reaction occurs (pH = 12), results in the chemical hydrolysis of 1-LG into LA and glycerol. Whether 13-HODE-G is hydrolyzed into 13-HODE under basic conditions has not been explored in depth to date. However, the detection of 13-HODE and LA in our reaction mixtures indicates that pH might be an important issue to consider, notably for the long-term storage of purified 13-HODE-G (and 13-HODE-G-d_5_). 

The results obtained here with human recombinant 15-LO-1 and -2 data (Figure 3) indicated that both enzymes metabolize 1-LG and LA to the same extent. Interestingly, when added simultaneously, LEA was preferentially metabolized over LA and 1-LG by either enzyme. This finding is consistent with those of our previous publication, in which we observed a 13-HODE-EA/13-HODE ratio of ~3.5 for 15-LO-1 and ~3 for 15-LO-2 [21], and is also in agreement with the data from Ivanof et al. who found that human 15-LOs better oxidized AEA over AA [43]. While Ivanof also documented a slight preference of 2-AG vs. AA, we did not observe such preference for 1-LG vs. LA, under the conditions utilized. Further enzymatic experiments (V_max_ and K_m_ calculations) would be a key addition to further characterize the substrate preference of 15-LO-1 and 15-LO-2. 

Human eosinophils and neutrophils biosynthesized 13-HODE-G in response to 1-LG in a time- and concentration-dependent manner (Figure 5 and Figure 7). This biosynthesis was dramatically enhanced by the serine hydrolase inhibitor MAFP in both cell types (Figure 4, Figure 6 and Figure 8). This was expected as we previously showed that the 15-LO metabolite of 2-AG, 15-hydroxy-eicosatetraenoyl-glycerol, was hydrolyzed to the same extent as 2-AG in both neutrophil and eosinophil suspensions, a phenomenon that was prevented by MAFP [36]. Herein, MAFP also diminished and or prevented the hydrolysis of 13-HODE-G and 1-LG by eosinophils and neutrophils (data not shown). This indicates that MAFP also interferes with the hydrolysis of both 1-LG and 13-HODE-G, and that preventing such a reaction increases the levels of these compounds *in cellulo* and possibly *in vivo,* as previously suggested [27,36]. 

Human eosinophils and neutrophils also biosynthesized 13-HODE-EA in response to LEA. This was not modulated by MAFP, in agreement with the inability of the latter to enhance the AEA-induced 15-HETE-EA biosynthesis [20]. However, different biosynthetic profiles were observed when comparing human eosinophils and neutrophils. Indeed, while LEA-stimulated eosinophils biosynthesized 13-HODE-EA and 13-HODE in a 5.3:1 ratio, LEA-stimulated neutrophils biosynthesized 13-HODE-EA and 13-HODE in 1:0.37 ratio (Figure 4 and Figure 6). The hydrolysis of 13-HODE-EA into 13-HODE is very unlikely the main cause of 13-HODE presence in LEA-treated neutrophils, as 13-HODE-EA was not hydrolyzed rapidly, with 60% remaining after 15 min. 13-HODE-EA was even more stable in our eosinophil preparation with 90% of the added 13-HODE-EA remaining after 15 min, although this difference might be the consequence of a lower cell concentration of eosinophils (10^6^ cells/mL) vs. neutrophils (5 × 10^6^ cells/mL). Another explanation for the presence of 13-HODE in these reactions is, therefore, that LEA is hydrolyzed into LA, and then metabolized into 13-HODE. However, the half-life of LEA and the production of LA was similar between neutrophils and eosinophils. Furthermore, when we incubated eosinophils with the 15-LO-1 specific inhibitor BLX-3887 and LEA, we inhibited the biosynthesis of 13-HODE-EA, but not that of 13-HODE, suggesting that the production of 13-HODE from LEA is not 15-LO-1-dependent. It was previously reported that LA can be oxidized into 13-HpODE, a precursor of 13-HODE, but this process was reported after longer incubations with LA than the 15 min time point we evaluated [44]. The incubation of eosinophils and neutrophils led to a small release of LA (150 and 30 pmol/million cells respectively) Thus, we cannot exclude that this 13-HODE production in LEA-treated neutrophils and eosinophils is the consequence of a non-enzymatic oxidation of LA nor can we exclude a potential role of the NAAA, which is not inhibited by MAFP. 

In experiments in which MAFP-treated human eosinophils were independently treated with LA, LEA, or 1-LG, they biosynthesized 13-HODE, 13-HODE-EA, and 13-HODE-G at a 4:1:1 ratio (Figure 4). When the experiments were repeated by simultaneously treating human eosinophils with a combination of LA, LEA, and 1-LG, the 13-HODE:13-HODE-EA:13-HODE-G ratio was 6:3:1 (Figure 8). This indicates that human eosinophils better metabolize LA vs. LEA and 1-LG. This finding was unexpected, as it is in sharp contrast with the data obtained with human recombinant 15-LO-1 (Figure 3). Interestingly, our kinetic experiments showed that in contrast to the LA-induced 13-HODE biosynthesis, the 1-LG-induced 13-HODE-G and the LEA-induced 13-HODE-EA biosynthesis were delayed by ~1 min (Figure 5). Such a delay was previously observed by our group when we compared the AA- and the 2-AG-induced leukotriene B_4_ biosynthesis in human neutrophils [13]. This raises the possibility that like 2-AG, 1-LG and LEA do not penetrate/diffuse into eosinophil cell membranes as rapidly as LA and that perhaps, a cellular membrane transporter is needed to shuttle 1-LG/LEA within the cells, compared to fatty acids. It could also indicate that 1-LG or LEA do not reach the cellular 15-LO-1-containing compartments as rapidly as LA.

Human neutrophils also biosynthesized 13-HODE-G and 13-HODE-EA in response to 1-LG and LEA, respectively in a concentration-dependent manner. As for human eosinophils, MAFP was key for the assessment of 13-HODE-G biosynthesis. Moreover, LA was also a better substrate for neutrophils compared to 1-LG and LEA. Aside from these similarities, we also observed major differences between eosinophils and neutrophils. First, neutrophils produced around 100 times less 15-LO metabolites compared to eosinophils, in agreement with our previous study assessing the biosynthesis of 15-LO metabolites from fatty acids and endocannabinoids [20]. Second, the biosynthesis of 13-HODE, 13-HODE-G, and 13-HODE-EA was very rapid, reaching a maximal accumulation at 15 s and slowly decaying overtime (Figure 7), in contrast to eosinophils in which a delay was observed for the biosynthesis of 13-HODE-G and 13-HODE-EA. Furthermore, the biosynthesis of these mediators was not inhibited by the 15-LO-1 inhibitor BLX3887 (Figure 9). This is perfectly in line with our earlier study in which eosinophil-depleted neutrophils (which were also utilized herein) biosynthesized 15-LO metabolites, a phenomenon that was not prevented by 15-LO-1 inhibitors and poorly prevented by NDGA [20]. But again, we cannot exclude that the biosynthesis of 13-HODE, 13-HODE-G, and 13-HODE-EA by neutrophils is the consequence of a non-enzymatic oxidation of LA, 1-LG, and LEA respectively. Given that 15-LO-2 inhibitors are not commercially available, we did not investigate their involvement. 

The binding/functional assays performed with a subset of endocannabinoidome-related receptors did not allow us to pinpoint which receptor is activated by 13-HODE-G, G, while we have previously shown that TRPV1 is a receptor for 13-HODE-EA [21]. Nonetheless, we found that 13-HODE is a good PPAR-α agonist, in agreement with a recent study from Kämmerer and colleagues [45]. This was also supported by the in silico analysis, where 13-HODE adopts a binding conformation in which the carboxylate group engages a network of stable H-bonds with the polar residue Ser280, Tyr314, His440, and Tyr464 sidechains, while the hydroxyl group is alternatively involved in a H-bond with Thr279 and Cys276 (Figure 11). The carboxylate H-bond network strongly contributes to the binding/activation of PPAR-α, an effect that is shared with canonical agonists, but hampered in 13-HODE-G, where the esterification by a glycerol moiety both deletes the negative charge and introduces steric clashes in the 13-HODE moiety arrangement. However, 13-HODE-G was found in almost all mouse tissues that we analyzed. Interestingly, we found that 13-HODE-G levels are reduced in the liver of *ob*/*ob* compared to *db*/*db* and lean mice [41], possibly suggesting its role in obesity-induced hepatic disorders (e.g., fatty liver and accompanying inflammation).

## 5. Conclusions

In conclusion, we have shown here that human eosinophils and neutrophils can metabolize 1-LG and LEA into the novel endogenous lipid mediators 13-HODE-G and 13-HODE-EA, respectively. This is the first study documenting the existence of 13-HODE-G and the ability of human neutrophils and eosinophils to biosynthesize this metabolite and its endogenous analogue, 13-HODE-EA. Deciphering the role of 13-HODE-G and 13-HODE-EA in heath and inflammatory diseases will be of interest, knowing that 15-LO-1 can be dysregulated in inflammation [46,47].

## Figures and Tables

**Table 1:** LC-MS quantification of 15-LO metabolites.

Lipid	Internal Standard Used	Q1 → Q3	Retention Time (Min)	LLOQ (Fmol)
13-HODE-d_4_	-	299.10 → 198.15	9.89	-
13-HODE-G-d_5_	-	358.00 → 261.30	8.76	-
13-HODE-EA-d_4_	-	326.30 → 66.20	7.72	-
13-HODE	13-HODE-d_4_	295.5 → 277.30	9.94	25
13-HODE-G	13-HODE-G-d_5_	353.20 → 261.2	8.80	25
13-HODE-EA	13-HODE-EA-d_4_	322.00 → 62.2	7.76	25

**Table 2:** ^1^H-NMR and ^13^C-NMR data of 13-HODE-G.

CARBON	13-HODE-G (2)			
N°	δ C (ppm)	δ H (ppm)	Features	J (Hz)
1	174.3	NA	NA	NA
2	34.1	2.35	t	7.5
3	24.8	1.67–1.47	2H, br m	
4	22.6	1.32	m	
5	28.9	1.32	m	
6	28.9	1.32	m	
7	28.9	1.32	m	
8	27.6	2.18	m	
9	132.8	5.44	m	
10	127.8	5.98	t	10.9
11	125.7	6.49	dd	15.2, 11.0
12	135.9	5.67	dd	15.2, 6.8
13	72.9	4.16	1H, overlapped m	
14	37.3	1.67–1.47	2H, br m	
15	29.7	1.32	m	
16	29.3	1.32	m	
17	25.1	1.32	m	
18	14.1	0.89	t	7
1’	70.3	3.93	1H, m	
2’	65.2	4.16	2H, overlapped m	
3’	63.3	3.65	2H, m	

See Figure 1 for carbon number.

**Table 3:** Levels of 13-HODE-G (in fmol/mg of tissue ± SEM) in the gastrointestinal tract of mice.

Duodenum	Jejunum	Ileum	Caecum
9746 ± 4360	4810 ± 2692	1165 ± 185	997 ± 317

**Table 4:** Affinity of 13-HODE-G and related compounds for human recombinant CB_1_ and CB_2_ receptors. Results are shown as the mean ± SD.

Compound	CB_1_ Receptor			CB_2_ Receptor		
	IC_50_ (µM)	Ki (µM)	Max Tested (% Displacement)	IC_50_ (µM)	Ki (µM)	Max Tested (% Displacement)
13-HODE	>10	>10	10 µM (7.56 ± 2.3)	>10	>10	10 µM (38.34 ± 5.4)
13-HODE-G	>10	>10	10 µM (18.90 ± 6.1)	>10	>10	10 µM (33.58 ± 21.3)
1-AG	1.32 ± 0.46	0.15 ± 0.05	10 µM (90.4 ± 1.3)	1.02 ± 0.04	0.16 ± 0.01	10 µM (75.52 ± 2.4)
1-LG	2.81 ± 0.85	0.31 ± 0.15	10µM (95.8 ± 16.4)	>10	>10	10 µM (28.45 ± 9.4)

**Table 5:** Effect of 13-HODE-G and related compounds on human recombinant TRPV_1_-mediated intracellular calcium elevation. Results are shown as the mean ± SD. ^1^ Desensibilization was assayed using 100 nM capsaicin.

Compound	Efficacy (% Max Response)	Potency (EC_50_ in µM)	Desensibilization ^1^ (IC_50_ in µM)
**1-AG**	58.9 ± 1.2	0.39 ± 0.03	1.1 ± 0.1
**1-LG**	46.5 ± 0.9	0.23 ± 0.02	2.7 ± 0.3
**13-HODE**	< 10	-	> 50
**13-HODE-G**	< 10	-	> 50

## Data Availability

The data presented in this study is contained within the article or supplementary material.

## Supplementary Materials

The following are available online at https://www.mdpi.com/article/10.3390/cells10092322/s1, Figure S1: Strategy to synthesize 1-LG-d_5_, 13-HODE-G and 13-HODE-G-d_5_, Figure S2: 1H-NMR in CDCl3 of 1-LG-d_5_, Figure S3: 13C-NMR in CDCl3 of 1-LG-d_5_, Figure S4: 1H-NMR in CDCl3 of 13-HODE-G, Figure S5: 13C-NMR in CDCl3 of 13-HODE-G, Figure S6: HSQC in CDCl3 of 13-HODE-G, Figure S7: COSY in CDCl3 of 13-HODE-G, Figure S8: 1H-NMR in CDCl3 of 13-HODE-G-d_5_, Figure S9: 13C-NMR in CDCl3 of 13-HODE-G-d_5_, Figure S10: HSQC in CDCl3 of 13-HODE-G-d_5_, Figure S11: COSY in CDCl3 of 13-HODE-G-d_5_.
