# Supplementary material for: Biosynthesis of the Novel Endogenous 15-Lipoxygenase Metabolites N-13-Hydroxy-octodecadienoyl-ethanolamine and 13-Hydroxy-octodecadienoyl-glycerol by Human Neutrophils and Eosinophils
Source: Cells. 2021 Sep 5;10(9):2322. doi: 10.3390/cells10092322 (PMC8470279; doi:10.3390/cells10092322)
Supplement: Supplementary file 1 [file cells-10-02322-s001.zip › cells-1351157-supplementary.pdf]

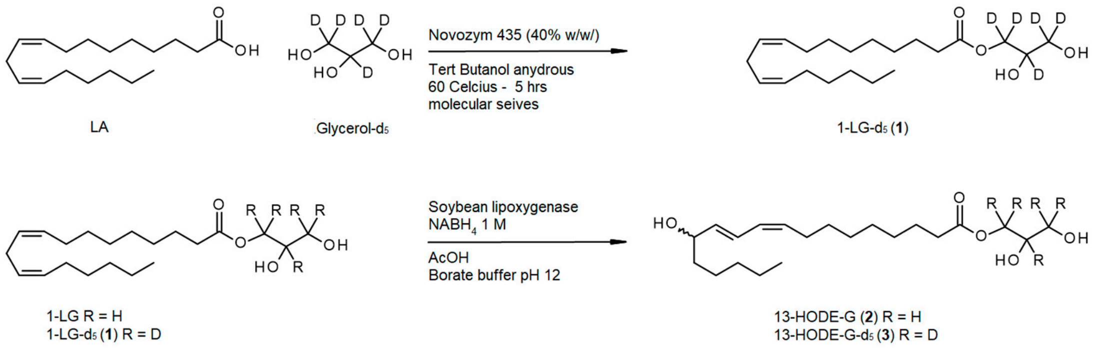

Figure S1. Strategy to synthesize 1-LG-d$_5$, 13-HODE-G and 13-HODE-G-d$_5$. LA and glycerol-d$_5$ were incubated with Novozym 435 to yield 1-LG-d$_5$. 1-LG or 1-LG-d$_5$ were then oxygenated by soybean lipoxygenase to yield 13-HODE-G and 13-HODE-G-d$_5$ respectively.

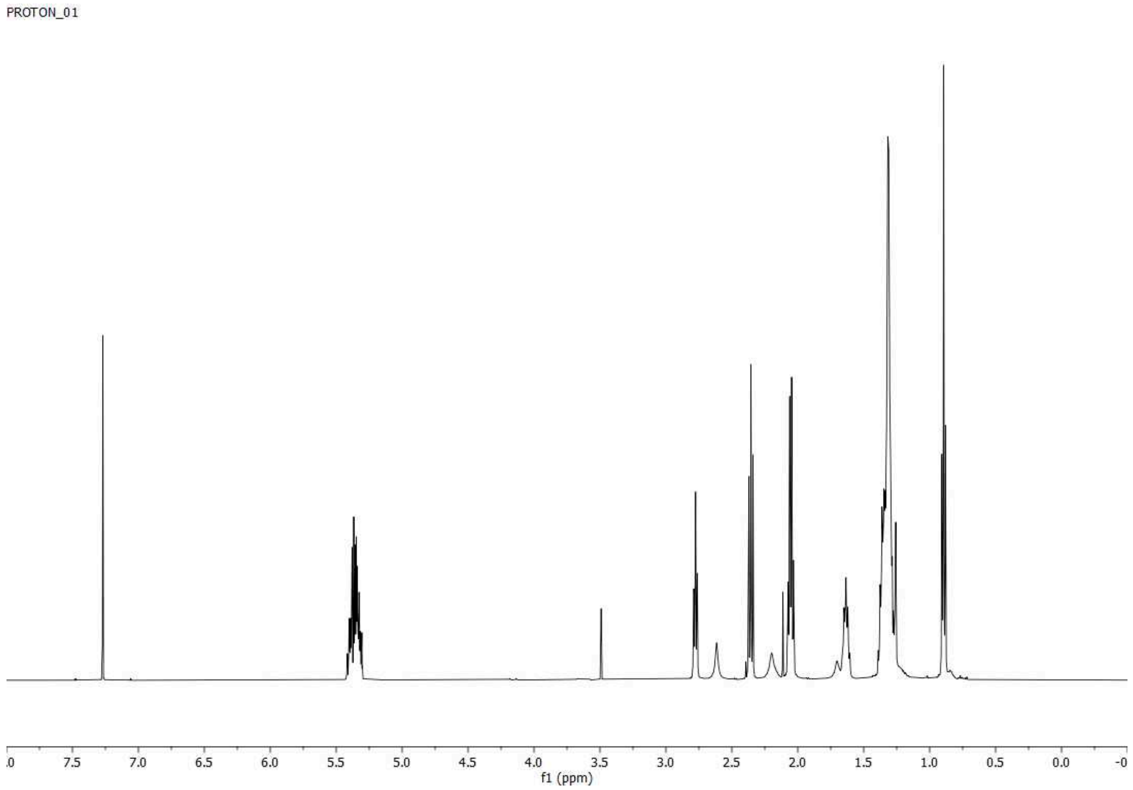

Figure S2. ^{1}H-NMR in $CDCl_3$ of 1-LG-d_5 (1).

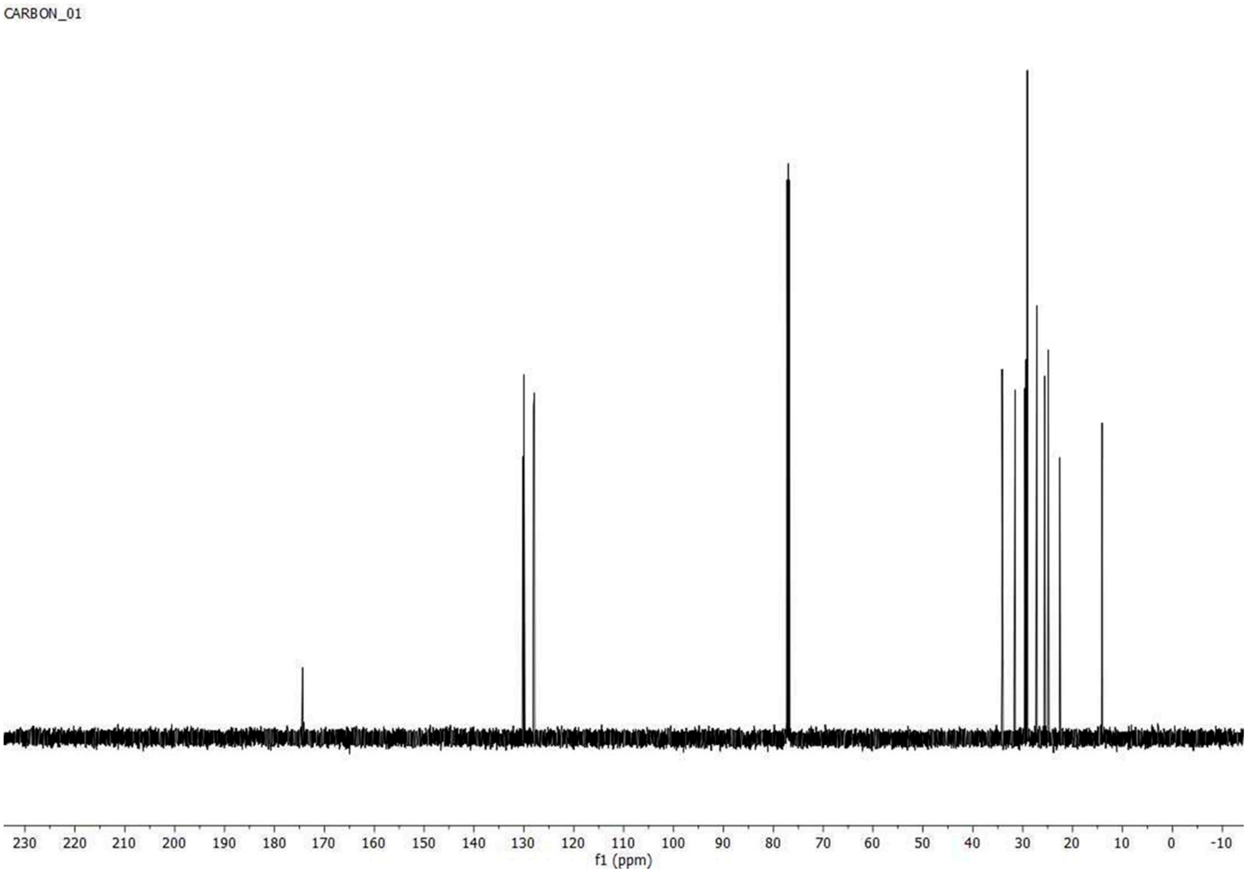

Figure S3. ^{13}C-NMR in $CDCl_3$ of 1-LG-d_5 (**1**).

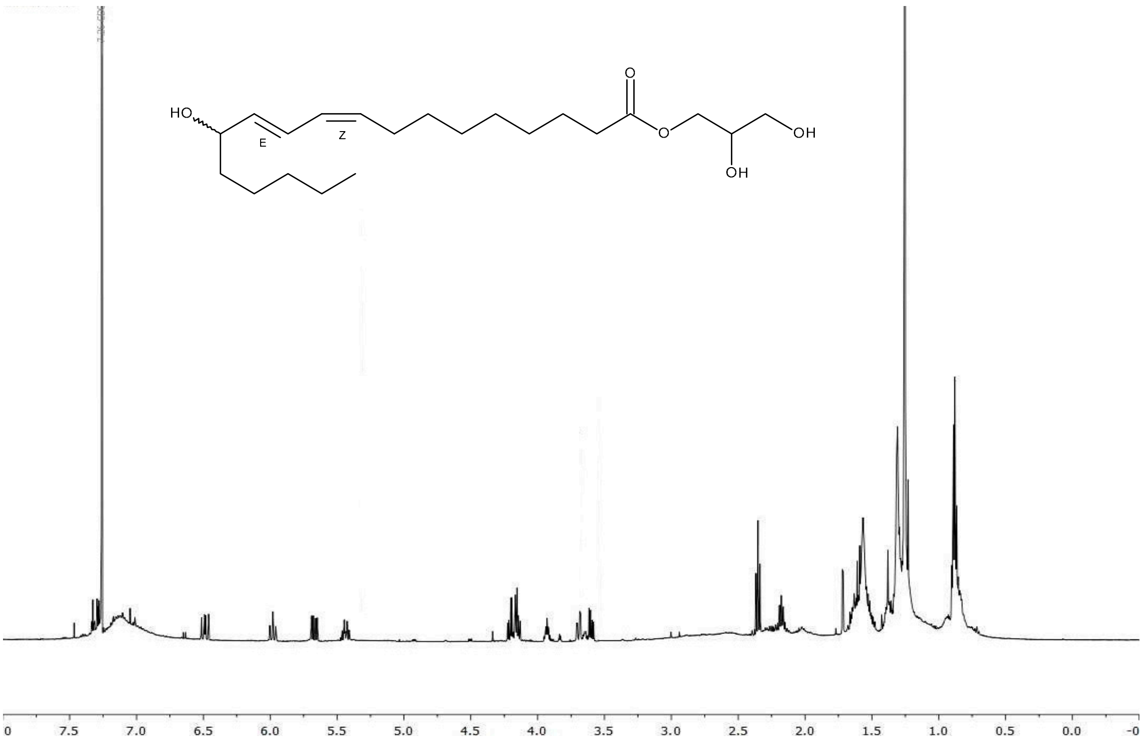

Figure S4. ^{1}H-NMR in $CDCl_3$ of 13-HODE-G (2).

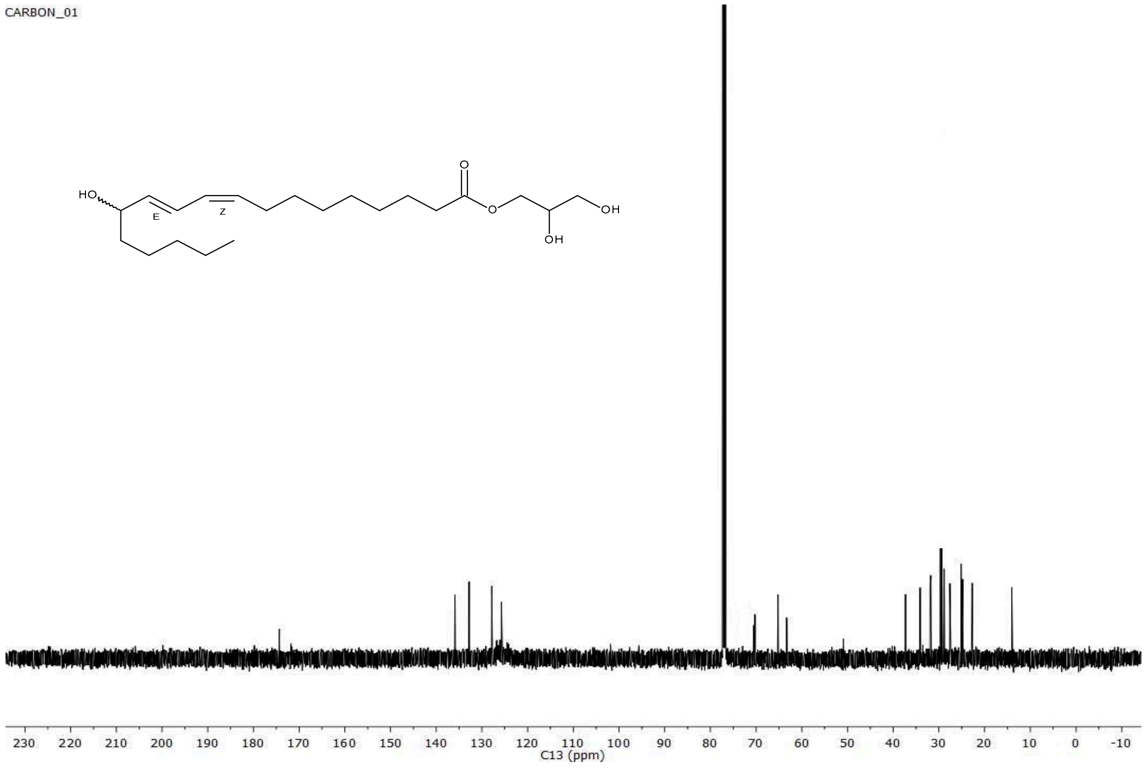

Figure S5. ^{13}C-NMR in $CDCl_3$ of 13-HODE-G (2).

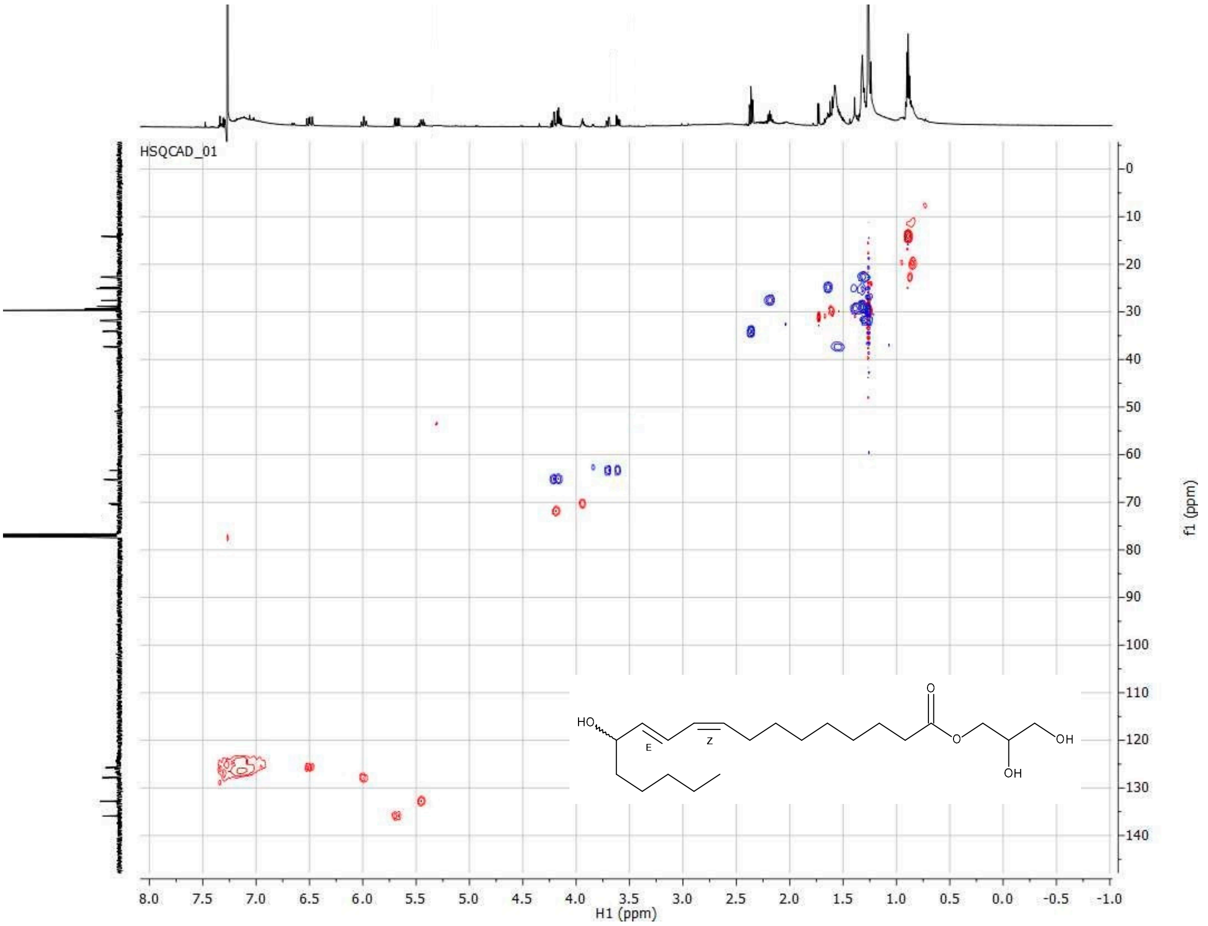

Figure S6. HSQC in CDCl3 of 13-HODE-G (2).

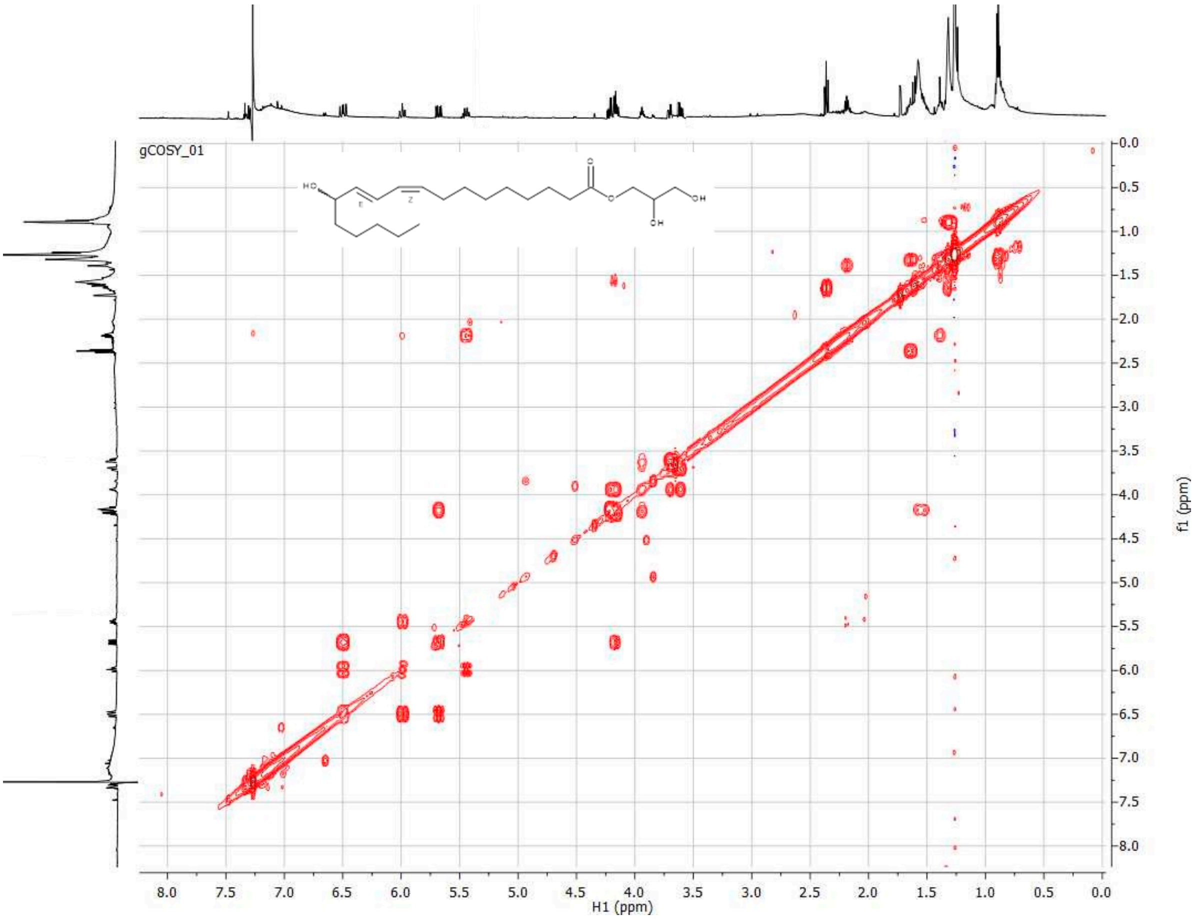

Figure S7. COSY in CDCl3 of 13-HODE-G (2).

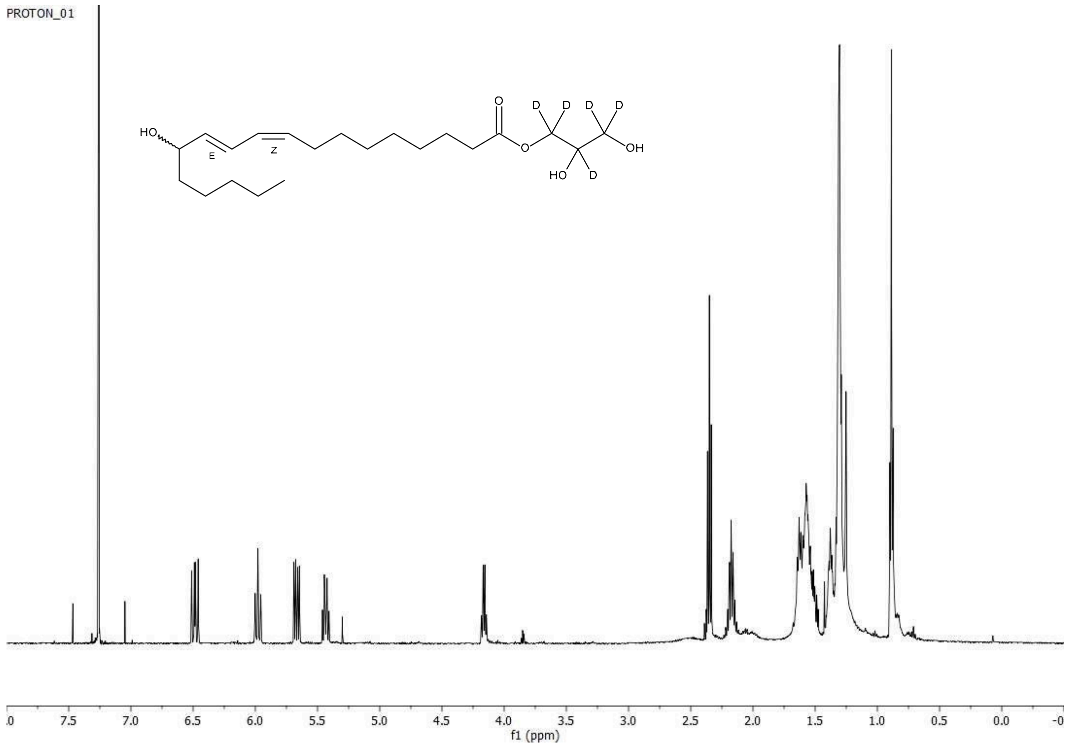

Figure S8. **^{1}H-NMR in $CDCl_3$ of 13-HODE-G-d_5 (3).**

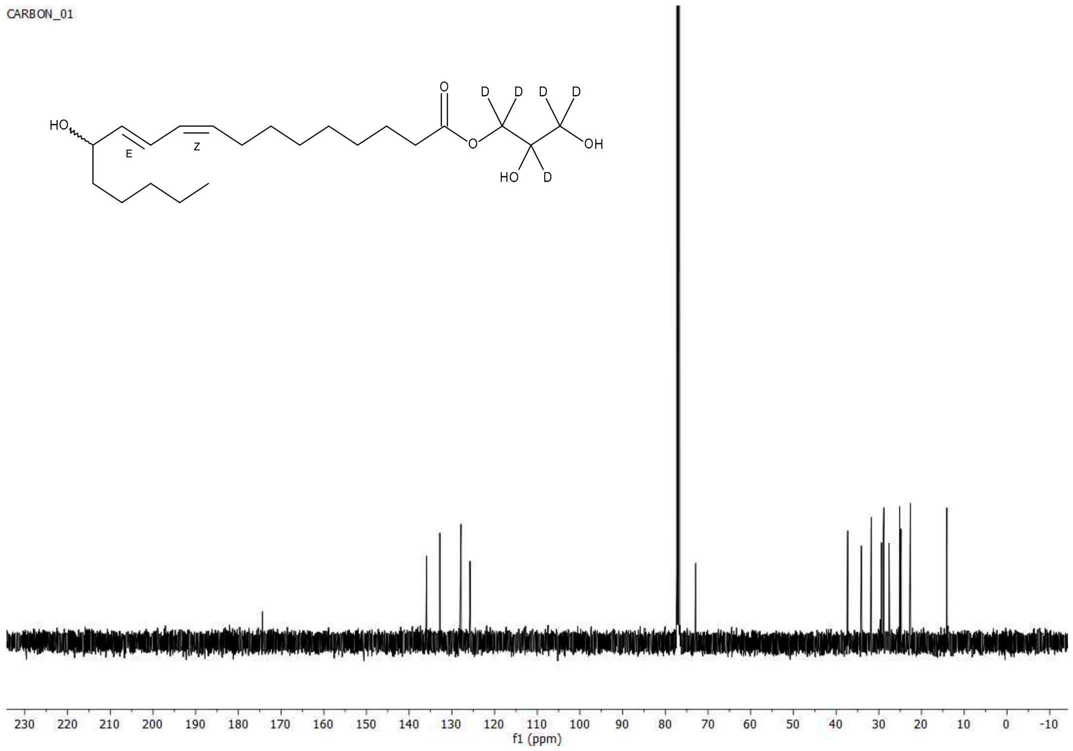

Figure S9. ^{13}**C-NMR in CDCl3 of 13-HODE-G-d$_5$ (3).**

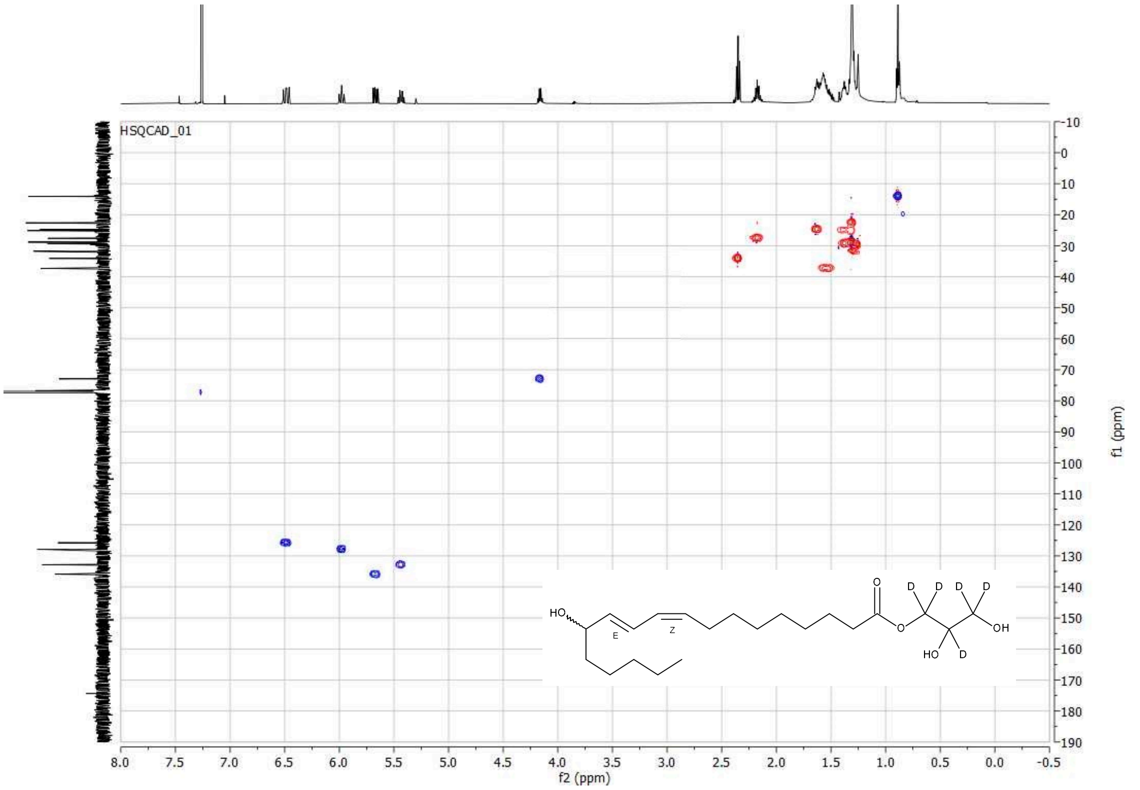

Figure S10. HSQC in $CDCl_3$ of 13-HODE-G-d_5 (3).

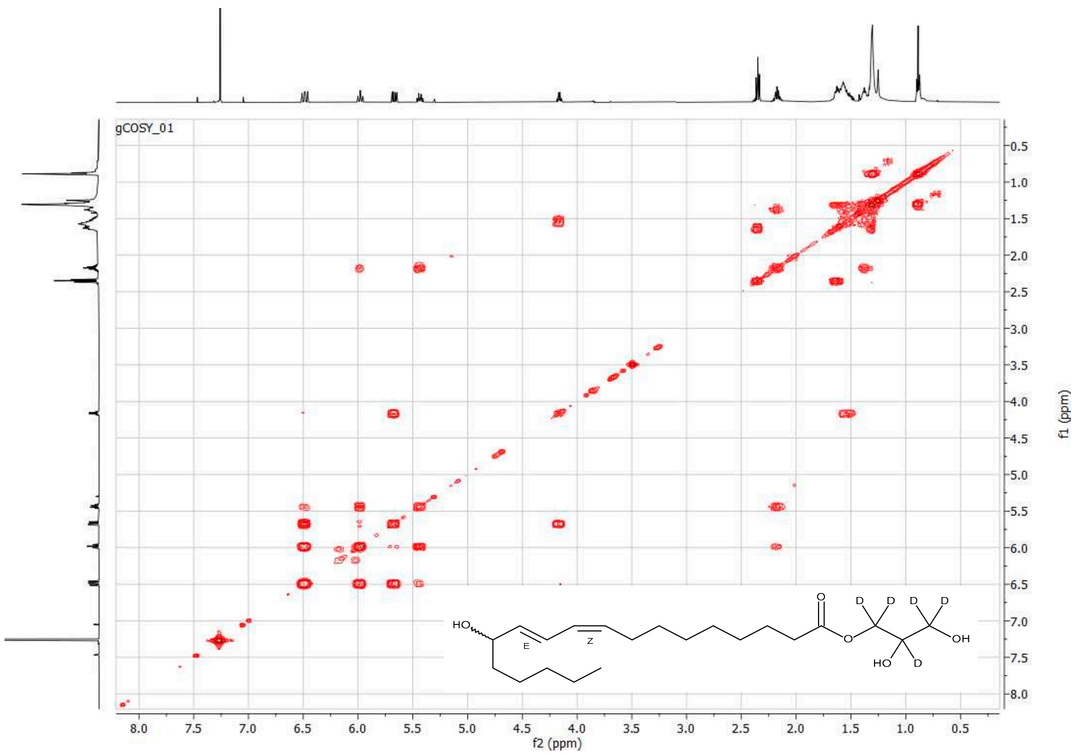

Figure S11. COSY in $CDCl_3$ of 13-HODE-G-d_5.
